# Clinical and Therapeutic Implications of Male Obesity

**DOI:** 10.3390/jcm12165354

**Published:** 2023-08-17

**Authors:** Monika Lenart-Lipińska, Michał Łuniewski, Joanna Szydełko, Beata Matyjaszek-Matuszek

**Affiliations:** Department of Endocrinology, Diabetology, and Metabolic Diseases, Medical University of Lublin, 20-954 Lublin, Poland; michael.luniewski@gmail.com (M.Ł.); jszydelko@interia.pl (J.S.); beata.matyjaszek-matuszek@umlub.pl (B.M.-M.)

**Keywords:** obesity, hypogonadism, male obesity, obesity treatment

## Abstract

The prevalence of obesity, a disorder linked to numerous comorbidities and metabolic complications, has recently increased dramatically worldwide and is highly prevalent in men, even at a young age. Compared to female patients, men with obesity more frequently have delayed diagnosis, higher severity of obesity, increased mortality rate, and only a minority of obese male patients are successfully treated, including with bariatric surgery. The aim of this review was to present the current state of knowledge about the clinical and therapeutic implications of obesity diagnosed in males.

## 1. Introduction

Nowadays, according to current recommendations, obesity should be considered a chronic, progressive, and relapsing disease with multifactorial pathogenesis that includes genetic, environmental, metabolic, and behavioral components [1]. Obesity is a treatable disorder and, if left untreated, is strongly associated with increased mortality and morbidity, including nearly two hundred complications such as cardiovascular disease, stroke, cancer, disability, type 2 diabetes, hypertension, sleep apnea, and many others [2]. It has been reported that up to 20% of adults live with obesity globally, and that overweight and obesity are the fifth leading cause of death in the world [3]. Furthermore, the prevalence of obesity is predicted to rise, especially in the developing countries. Many studies have shown that obesity affects people at any age, all geographies, and all socioeconomic backgrounds and is becoming more prevalent in men [4]. The World Obesity Atlas 2023, published by World Obesity Federation, predicts that more than half the global population will be living with overweight and obesity by 2035 if current trends continue [5]. What is particularly frightening is that obesity among children is growing faster than among adults. Projections estimate a 100% increase in the prevalence of overweight and obesity among boys and more than double that among girls (125% increase). If current trends prevail, one in four people worldwide will be overweight or obese. We strongly need prevention, treatment, and support to treat this dangerous disease, which is still seen by many as a purely aesthetic problem [5].

It has been observed that obesity can present differently in women than in men [6]. Women show the metabolically healthy obese phenotype more often than males. Due to hormonal differences, women tend to have more subcutaneous fat, which decreases insulin resistance, while men have more visceral and liver fat, which results in higher cardiovascular risk. Obese women are more prone to suffer from weight-related problems, have a higher rate of depression, and tend to seek medical attention to manage obesity earlier than men. Compared to female patients, men with obesity more frequently have delayed diagnosis, higher severity of obesity, an increased mortality rate, and only a minority of obese male patients are successfully treated, including with bariatric surgery [7].

Neuroimaging studies have highlighted several potential mechanisms responsible for individual susceptibility to obesity, including sex differences [8]. There is a significant body of evidence that obesity is associated with an abnormal structure, function, and chemistry in the brain’s reward system, especially confirmed in obesity among female patients. It has been established that women demonstrated greater neural responses in striato-limbic and frontal-cortical regions in response to food cues than men [9]. In men, changes in somatosensory regions were found to be linked with obesity phenotype and total body fat was negatively correlated with gray matter volume in subcortical regions (including the thalamus, caudate nucleus, putamen, hippocampus, and the nucleus accumbens) [10]. Although many discrepancies between studies exist, understanding these neural and behavioral sex differences may be crucial for personally tailored approaches, including lifestyle interventions for the prevention and management of obesity. Therefore, the treatment of obesity should be team-based and, above all, individualized.

Women and men perceive obesity differently. Obese men tend to display lower self-esteem to a lesser extent than women and do not consider obesity a disease. It may be the underlying reason why the literature on male obesity is lacking, especially when compared to publications covering the diagnosis and treatment of obesity in women. Furthermore, it should be recognized that male obesity is a topic that requires an in-depth analysis, not only in purely medical terms, but also in economic, social and psychological aspects. Previous studies have rarely referred to the comparison of android-type versus gynoid-type obesity or to the comparison of testosterone-to-estrogens ratio on the phenotype and the course of obesity. It is noteworthy because, as evidenced by numerous scientific studies, obesity treatment should be cause-dependent if possible. This means that obesity management should preferentially target the individual-based pathogenetic mechanisms behind the development of obesity.

This article reviews clinical and therapeutic implications of obesity with an emphasis on male-related aspects of the disease.

## 2. The Psychological Aspect of Obesity and the Influence of Cortisol

Obesity has its imprint on broadly defined health since adolescence, when the complicated social and interpersonal relationships combine with various physiological processes related to sexual maturation [11,12,13]. Both of the above-mentioned factors are known stressors, and their stressogenic effect is even more pronounced in the case of coexistence of obesity as a cause of stigmatization and discrimination in the peer group [14]. This is, for instance, due to the increased activity of the HPA (hypothalamic-pituitary-adrenal) axis in puberty [15]. The anxiety and feeling of danger associated with social ostracism activates the HPA axis, which results in an increase in the cortisol secretion, a hormone that plays a leading role in chronic stress reactions. These high levels of cortisol are, in turn, associated with the development of Cushing’s syndrome. One of its most pathognomonic features is central obesity, that is trunk localized obesity, with a simultaneous muscle mass wasting, manifested by thinning of the limbs. Such a change in physique only fuels further stigma, giving rise to a vicious cycle mechanism [16]. Other unfavorable effects of cortisol include decreased fertility and disturbances in carbohydrate metabolism in the form of hyperglycemia (both increased gluconeogenesis and glycolysis) and insulin resistance [17,18,19].

Long-term stress stimulation secondary to hypercortisolemia strongly upsets the whole homeostasis, establishing a new, incorrect set point. The body is highly reactive to all forms of stress and the affected individual adapts to it by establishing incorrect eating habits. The patient chooses palatable but unhealthy food and has a tendency to over-eat or under-eat [20]. In the long term, increased cortisol levels translate into an increased risk of type 2 diabetes mellitus, metabolic syndrome, and cardiovascular complications [21,22].

As mentioned above, stigma increases the feeling of stress. The patient tries to get rid of this tension through stress-reducing behaviors. Paradoxically, in people suffering from obesity, the primary way to reduce stress involves overconsumption of palatable foods. This, in turn, results in a quick, though short-term, improvement in mood [23], followed by the increase in body weight and an eventual exacerbation of the discrimination-related problems [24]. Should this type of behavior occur in adolescence, its persistence into the future—as a disturbance in eating and mood-improving behavior—is very likely [25]. It may also result in future eating disorders in the form of anorexia nervosa or bulimia nervosa [26].

Research shows that androgens are not as closely associated with increased depression in chronic stress states as female sex hormones are [27,28]. Moreover, androgens do not induce eating disorders, such as anorexia nervosa or bulimia nervosa, in contrast to estrogens, which display such effects, hence the rare occurrence of eating disorders in males [29]. However, there are data indicating that lower testosterone in men is associated significantly with eating disorders regardless of depressive symptoms, body mass index, and age [30].

## 3. Male Obesity Overview

Men are less willing to treat their obesity when compared to women. This applies to behavioral treatment involving a change in lifestyle e.g., physical activity, dietary treatment. This is also relevant to pharmacological and surgical therapy.

It has been believed that the reason for this fact is that men are less likely to pay attention to their external appearance and generally focus less on health problems. Moreover, when compared to women, male eating habits are more often unhealthy and males exhibit less knowledge about proper nutrition [31,32].

Android-type obesity, typical for men, as the name itself implies, is characterized by the overaccumulation of adipose tissue in the trunk area. Hence, the name “central obesity”, or “apple-shaped” obesity. Central obesity is more readily associated with the coexistence of visceral obesity. Both types of obesity, central and visceral, are strictly linked to each other and strongly contribute to the occurrence of obesity-related cardiovascular complications [33,34,35].

Visceral adipose tissue is considered a very active endocrine organ, secreting numerous molecules such as adipokines, growth factors, and hormones. These chemical mediators, in turn, promote and exacerbate insulin resistance and induce chronic inflammation.

Alterations in the secretion and function of adipokines, signaling molecules secreted by adipose tissue, playing crucial roles in regulating numerous processes, including metabolism, inflammation, and immune responses, have been associated with the imbalance in various pathways involved in the pathogenesis of obesity [36]. The main adipokines associated with development of obesity are leptin, adiponectin, resistin, TNF-alpha, and IL-6 [37]. Leptin regulates energy balance and acts on the hypothalamus, suppressing appetite and increasing energy expenditure. In obesity, there is leptin resistance, resulting in decreased sensitivity to its signals, which can lead to overeating and weight gain. Leptin levels are generally higher in females than in males, which may be explained by greater proportion of subcutaneous adipose tissue when compared to males. Higher leptin levels in females have been suggested to contribute to greater leptin sensitivity, potentially providing a protective effect against obesity [38].

Adiponectin is another important adipokine secreted by adipose tissue, playing a key role in improving insulin sensitivity, enhancing fatty acid oxidation, and reducing inflammation [39]. In obesity, adiponectin levels tend to decrease, which is associated with insulin resistance, dyslipidemia, and a pro-inflammatory state. Females typically have higher circulating adiponectin levels when compared to males, which may partly contribute to increased insulin sensitivity and lower risk of metabolic disorders compared to males [40].

In obesity, there are observed higher levels of resistin, contributing to impaired insulin signaling and increased inflammation, which can further worsen metabolic dysfunction. In some studies, males have been found to have higher resistin levels when compared to females, potentially contributing to their higher susceptibility to insulin resistance and metabolic disturbances [41].

TNF-alpha and IL-6 are pro-inflammatory cytokines that can be produced by adipose tissue in response to obesity-related stress [42]. Elevated levels of these molecules can promote insulin resistance and contribute to chronic low-grade inflammation. Males tend to have higher circulating levels of pro-inflammatory cytokines when compared to females, which may partly explain their higher prevalence of obesity-related inflammatory diseases [43].

The observed differences in adipokine concentrations between males and females may contribute to variations in fat distribution and metabolic responses. Understanding these sex-specific differences in adipokines can have implications for developing personalized approaches to obesity prevention and treatment and may help in understanding the different health risks associated with obesity between men and women. Further research is needed to fully elucidate the underlying mechanisms and implications of sex-specific differences in adipokines profile in obesity.

In the course of obesity, concentrations of proinflammatory cytokines in the circulation are observed to rise due to infiltration and activation of macrophages in the metabolically-altered visceral adipose tissue [44]. In the experimental studies, it was determined that chronic inflammation is considerably more marked in obese males than females. Male adipose tissue is infiltrated by higher numbers of macrophages, and these macrophages were found to be more inflammatory and have higher propensity to be migratory [45]. The migration of macrophages is stimulated in response to increased leptin levels, which were significantly higher in males with obesity in comparison to females. These observations may partially elucidate sex differences in obesity-associated diseases which contribute to much more increased cardiovascular risk in obese males than females.

## 4. Pathogenetic Factors of Obesity

There are many factors that contribute to the pathogenesis of obesity, including genetic, environmental, social, and cultural factors. They have a similar effect on the risk of obesity development in both sexes: males and females. Changes in eating habits e.g., easy access to palatable food or the widespread availability of fast food, play an important role, as do genetic factors. In the case of genes, multi-gene interactions play far more important role in promoting the obesity phenotype than those associated with isolated gene mutations since monogenic obesity is rare. So far, over 100 different gene polymorphisms responsible for obesity have been identified. Their individual contribution to emergence of obesity is small; however, the coexistence of numerous mutations synergistically increases the risk of obesity to a great extent. The strongest association with obesity was found for the FTO (the fat mass and obesity associated gene) [46].

The most common form of monogenic obesity is caused by a mutation of the gene MC4R, a gene responsible for suppression of the perception of appetite. This form of monogenic obesity accounts for about 5% of obesity diagnosed in pre-adults [47,48]. A new drug, setmelanotide, an MC4R agonist, shows very promising results in the treatment of this type of obesity. It is a drug that, through the activation of the melanocortin pathway, acts on the underlying cause of the disease [49]. Therefore, this is a rare case where the treatment of obesity is directed strictly at the very pathologic basis of the obesity.

Other causes of monogenic obesity, such as POMC (proopiomelanocortin) gene mutations or leptin gene mutations, are much rarer. Furthermore, these types of monogenetic obesity take a more extreme course, and are characterized by unstoppable and uncontrollable hyperphagia [50,51]. Similarly, genetic syndromes (Prader–Willi syndrome, Bardet–Biedl syndrome) are rare (1: 15,000–100,000 live births, depending on the syndrome) [52].

The adipose tissue itself also plays an important role in both protecting against obesity and promoting its occurrence. The net result depends on the types of substances secreted by adipocytes. The adipocyte-derived substances participating in the regulation of the metabolism of adipose tissue and therefore determining the occurrence of obesity include, among others: adipokines and hormones (leptin, resistin, adiponectin, estrogens), cytokines (interleukins 1, 6, 8, 10 etc.), other substances, e.g., enzymes (cholesterol ester transfer protein, lipoprotein lipase), and many more [53].

A significant risk factor for obesity in adulthood is obesity diagnosed in childhood or obesity diagnosed in one of the parents. It is currently unknown whether the cause of this relationship is related to childhood conditioning or whether it is due to genetic and epigenetic factors.

## 5. Clinical Consequences of Male Obesity

The increasing prevalence of obesity, considered an unsolved problem of public health, is linked with long-term metabolic consequences, such as cardiovascular disorders, type 2 diabetes mellitus, atherogenic dyslipidemia, various types of cancer, stroke, obstructive sleep apnea, obesity hypoventilation syndrome, and metabolic associated fatty liver disease (MAFLD, formerly known as NAFLD, non-alcoholic fatty liver disease). Obesity complications specific to men include hypogonadism, erectile dysfunction, and infertility.

### 5.1. Obesity, Hypogonadism and Infertility in Men with Obesity

In many epidemiological studies the positive correlation between obesity and male hypogonadism has been observed, indicating increased adiposity as the primary reason standing behind the gonadal dysfunction. Furthermore, in other obesity-related disorders, such as type 2 diabetes mellitus, components of metabolic syndrome, hypogonadism has been frequently reported. It is worth noting that there is a bidirectional relationship between obesity and hypogonadism as obesity may be a cause of hypogonadism, whilst low testosterone levels, by affecting proper body composition, may give rise to obesity. However, findings published by Cheung et al., pertaining to men undergoing androgen deprivation therapy for prostate cancer, indicate that significantly reduced circulating testosterone levels have only a minor effect on body weight. Thus, it may be concluded that increased adiposity may exert a more potent effect on the suppression of gonadal axis rather than the other way around [54]. Additionally, the commonly observed remission of hypogonadism with weight loss indicates a crucial role of obesity in the pathogenesis of gonadal dysfunction [55].

Male obesity is linked to hormonal disturbances leading to impaired gonadal functions, both hormonal and reproductive. It has been observed that almost 20% of the subfertility and infertility cases in males can be attributed to overweight and obesity [56]. Male obesity-related hypogonadism has been reported in up to 45% of patients with moderate to severe obesity, based on the decreased plasma testosterone concentrations, measured according to good practice procedures (that is in the morning and at a fasting state), combined with typical signs and symptoms of hypogonadism [57]. Many studies have confirmed that in obese males, androgen levels decline in proportion to the degree of obesity.

The causative mechanism of obesity-induced hypogonadism is yet to be defined but is likely multifactorial.

Hypogonadism in obesity may develop in several mechanisms, disrupting the hypothalamic-pituitary-testicular (HPT) axis function. Investigation of available literature suggests that estradiol, leptin, and adipokines secreted by visceral adipose tissue are the crucial players in obesity-associated hypogonadism in men. The role of estradiol has been elucidated in many studies. This estrogen is secreted by the testes and adipose tissue after undergoing conversion from testosterone, a reaction catalysed by aromatase. Estradiol is responsible for negative feedback loop and suppression of the HPT axis in men, mainly at the level of the pituitary gland [58]. Many observations have corroborated that estradiol concentrations are often increased in men with obesity, which can be explained by increased aromatase-dependent testosterone conversion into estradiol in peripheral tissues [59]. Due to increased adiposity, aromatase expression is proportionally higher in males with obesity, which in turn results in elevated estradiol concentrations and, consequently, in the suppression of the HPT axis. Thereupon, a decrease in testosterone secretion in the testes is observed. These findings are supported empirically by the fact that the hypogonadism due to the suppression of HPT axis is reversible upon weight loss [55,60]. After significant weight reduction, an increase in gonadotrophins and testosterone, as well as lower estradiol concentrations were observed. Furthermore, Loves et al., in the further corroboration of the aforementioned observation, demonstrated that treatment with aromatase inhibitors induced the same changes in hormonal concentrations as weight loss in hypogonadal men with obesity [61]. Contrary to these data, Dhindsa et al. reported lower total and free estradiol concentrations and subnormal testosterone concentrations in a subpopulation of obese men with type 2 diabetes mellitus when compared to the control group without biochemical hypogonadism. The researchers concluded that the suppression of the HPT axis in patients with subnormal free testosterone concentrations and type 2 diabetes mellitus was not associated with increased estradiol concentrations and that the pathogenesis of subnormal free testosterone concentrations in type 2 diabetes mellitus needs further investigation [62,63]. Other authors emphasize the role of aromatase polymorphisms that positively correlate with elevated estradiol concentrations and obesity [64]. Remarkably, a decrease in estradiol concentration was followed by weight loss only in the patients with confirmed polymorphism. Moreover, Ghanim et al. found lower expression of aromatase and diminished androgen and estrogen receptors density in patients with type 2 diabetes mellitus coexistent with hypogonadism [65]. On the other hand, the limitation of immunoassays credibility, especially while interpretating lower estradiol results, should be taken into consideration when hypogonadism is suspected. Huhtaniemi et al. confirmed that immunoassays used for estradiol measurements may only be suitable for the detection of high estradiol concentrations in men [66]. In conclusion, the exact estradiol role in the pathogenesis of hypogonadism in obese men requires further investigation.

Another key player involved in obesity-related hypogonadism is leptin, an adipokine predominantly secreted by white adipocytes and involved in promotion of satiety and energy expenditure [67]. The identification of leptin and its receptors, along with studies performed in animal models of leptin deficiency and leptin resistance, have connected the role of leptin in broadly understood reproduction and revealed new aspects of the relationship between energy stores, adipose tissue, and reproductive function. Leptin has been reported to be an important regulator of male fertility by regulating the HPT axis thorough the stimulation of the hypothalamus via kisspeptin to secrete and release GnRH. In experimental studies, leptin administration improved pulsatile secretion of GnRH in rodents [68]. Obesity leads to leptin resistance and alters its normal functions [69]. There is a great body of evidence that most common forms of obesity are associated with excessive circulating levels of leptin, secondary to leptin resistance. In tandem with the progression of obesity resultant from increased adipose tissue mass, an accompanying significant increase in circulating levels of leptin is observed and correlates positively with total body weight and adiposity. Isidori et al. demonstrated that circulating leptin was the hormonal factor most closely associated with low testosterone and inversely correlated with testicular response to hCG stimulation. In the course of leptin resistance, the suppression of kisspeptin gene expression and kisspeptin receptors occurs in the HPT axis with a subsequent decrease in GnRH and gonadotrophin release and consequent decrease in testosterone secretion [70]. Besides hypothalamic influence, leptin exerts its action at the testicular level. It has been reported that high leptin concentration in obesity inhibits Leydig cells function and, thus, testosterone synthesis [71]. Curiously, testosterone alone inhibits leptin secretion from adipocytes and decreases circulating leptin levels independently from changes in the adipose tissue cells [72,73].

This observation allows us to speculate that testosterone replacement therapy may be beneficial to restore proper response of the HPT axis to leptin. However, further investigation into the matter is required to establish future therapies for male infertility. According to recent guidelines, testosterone administration can be considered only individually, that is on a case-by-case basis, after carefully weighing potential benefits, side effects, and risks if weight loss cannot be achieved and clinical and biochemical hypogonadism persists [74].

It is well established that higher adiposity, leading to oversecretion of adipokines and proinflammatory cytokines by visceral adipose tissue, also affects fertility in men. Obesity has numerous negative effects on sperm quality e.g., spermatozoa motility and vitality. In the meta-analysis by Sermondade et al., increased prevalence of azoospermia or oligospermia in obese males was confirmed [75].

**Figure 1:**
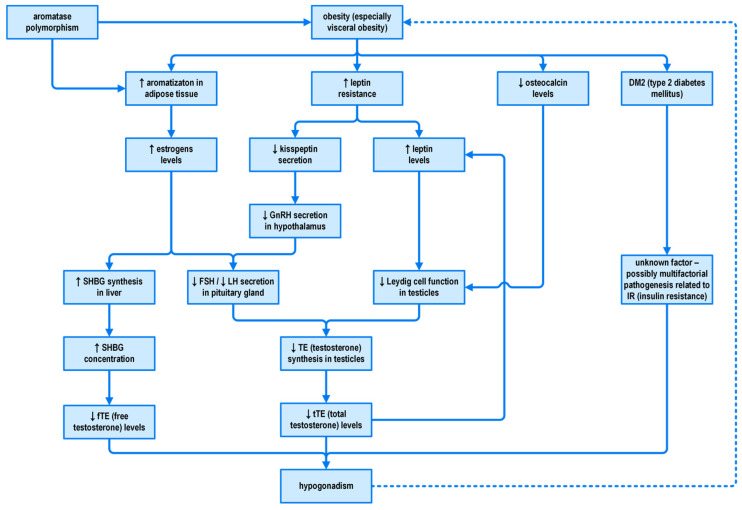
The pathogenesis of hypogonadism in male obesity. SHBG—sex hormone-binding globulin; GnRH—gonadotropin-releasing hormone; FSH—follicle stimulating hormone; LH—luteinizing hormone; ↑—an increase; ↓—a decrease; a dashed line means weak relationship.

It needs to be highlighted that obesity is associated with hypogonadism and fertility difficulties through many mechanisms. Contrary to other types of hypogonadism, obesity-related disturbances are usually functional in nature and therefore, potentially reversible, if proper treatment is commenced. It should be emphasized that weight loss in obesity is thought to be a key intervention that must be undertaken in order to restore HPT axis hormonal imbalances. Considering the long-haul effects of male obesity treatment in the broad context of gonadal axis function, it is of paramount importance that weight loss results in elevation, or more precisely, normalization of both total and free testosterone, FSH, and SHBGand a decrease in estradiol and prolactin levels [76]. In addition, beneficial results are seen after reduction of the amount of visceral tissue, which—as stated before—is rich in aromatase. Reduction in visceral adipose tissue with the concomitant decrease in aromatase activity is responsible for the lowered ratio of testosterone to estradiol conversion. This in turn allows for the improvement of male obesity secondary to hypogonadism [77,78,79]. Experimental studies have also shown that a decrease in BMI (body mass index) leads to the increase in the levels of osteocalcin; osteocalcin directly stimulates Leydig cells in the testes to synthesize testosterone [80]. The observed improvement in the hormonal and metabolic profile due to weight reduction along with other beneficial changes in the body such as reduction of endothelial dysfunction facilitates reversal of erectile dysfunction [81]. The pathogenesis of hypogonadism in male obesity is illustrated in Figure 1.

### 5.2. Diabetes Mellitus and Cardiovascular Disorders

It has been widely recognized that complex crosstalk exists between body composition, androgen levels, obesity, hypogonadism, type 2 diabetes mellitus, and vascular disease in men. Obesity is considered to be a major risk factor in the development of diabetes mellitus and may precede overt type 2 diabetes mellitus for many years. Taking into consideration that type 2 diabetes mellitus and other obesity-related metabolic consequences, such as insulin resistance, atherogenic lipid profile, and hypertension, promote and exacerbate atherosclerosis, each should be treated as a cardiovascular disorder. It has been well established that a reduction of 5–10% of body weight results in improvement of cardiovascular disease (CVD) risk.

Observational analysis of participants in the Look AHEAD (Action For Health in Diabetes) study (*n* = 5145; 40.5% male, 37% from ethnic/racial minorities) conducted by Wing et al. confirmed that modest weight losses of 5 to 10% were associated with significant improvements in CVD risk factors at year 1. The larger the weight loss, the greater the benefits [82].

In addition, intensive lifestyle interventions produce clinically meaningful weight loss (≥5%) at year 8 in 50% of the patients with type 2 diabetes mellitus and, thus, can be used to manage other obesity-related comorbidities [83].

### 5.3. Cancer

Obesity increases the risk of several types of cancer and is associated with poorer outcomes when compared to general population. The link between obesity and increased cancer incidence and cancer-related deaths has been well established over the last two decades, and it has been estimated that 14% of cancer deaths in men and 20% in women are attributable to obesity [84]. Calle et al. observed a significant positive linear trend in death rates in men with increased body-mass index for all cancer types, including esophageal, stomach, colorectal, liver, gallbladder, pancreatic, prostate, and kidney cancer, as well as non-Hodgkin’s lymphoma, multiple myeloma, and leukemia. A growing body of evidence indicates that, at the cellular level, obesity-related low grade chronic inflammation is a major cancer promoting event, leading to changes in immune cell infiltration and polarization in white adipose tissue [85]. Another hypothesis states that obesity results in elevated levels of insulin and insulin-like growth factor 1 (IGF-1), a known mitogen [86]. Binding of IGF-1 to its receptor leads to activation of intracellular signaling pathways, promoting proliferation and, in the long run, oncogenesis.

Other emerging studies put the role of elevated leptin and decreased adiponectin levels in obese patients in the spotlight of cancer initiation and promotion. It is increasingly evident that dysregulation in leptin and adiponectin balance is a key player in obesity-associated cancer development and progression. Leptin is known to be protumorigenic and proangiogenic, and to act directly on cancer cells to promote their proliferation and inhibition of programmed cell death [87]. Adiponectin has been shown to be antitumorigenic and, consequently, hypoadiponectinemia is associated with increased tumor growth [87].

The molecular mechanisms triggering cancer development in obesity have been elucidated to some extent. However, many mechanisms are still not well understood and therefore remain the focus of intensive research.

### 5.4. Metabolic Associated Fatty Liver Disease (MAFLD)

MAFLD, a liver condition characterized by the accumulation of fat in the liver due to metabolic dysregulation, is closely connected with obesity. However, other factors, such as genetics, lifestyle, and underlying health conditions, play significant roles in its development. Both males and females can develop this complication. As such, some studies suggest that males may have a higher prevalence of MAFLD when compared to females [88]. This difference may be partly attributed to hormonal variations and distribution of body fat.

Obesity is a well-established risk factor for MAFLD. Abdominal obesity, more pronounced in men, is strongly positively associated with a higher prevalence of MAFLD. Additionally, central obesity is closely linked to insulin resistance which leads to increased fat accumulation in the liver, contributing to MAFLD development. Abdominal obesity triggers a chronic low-grade inflammatory state not limited to fat tissue, also affecting the liver. The combination of inflammation and fatty liver can progress to more severe stages of MAFLD, such as non-alcoholic steatohepatitis (NASH) and cancer [89].

Besides obesity, MAFLD is associated with other metabolic risk factors such as type 2 diabetes, atherogenic dyslipidemia, and hypertension. In men with obesity, these metabolic disturbances are more prevalent, further contributing to the development and progression of MAFLD. MAFLD in obese men can lead to serious health complications, including advanced liver fibrosis, cirrhosis, and an increased risk of cardiovascular disease.

Given the strong association between obesity and MAFLD in men, addressing obesity through lifestyle modifications remains the cornerstone of MAFLD management, and any medical intervention should be tailored to the individual’s specific needs and health status.

Early detection and proper management of comorbidities can help reduce the impact of MAFLD on cardiovascular risk and life expectancy. There is no specific pharmacotherapy approved solely for the treatment of MAFLD. However, pioglitazone ad SGLT2 inhibitors have been studied for their potential benefits in managing MAFLD [90,91].

## 6. Obesity Treatment

After obesity diagnosis, the patient should be screened for obesity complications and obesity therapy should be commenced without further ado.

Obesity management ought to be based on its causes (if possible) and both the severity of the disease and its complications should be taken into account in order to find the most suitable therapy. This custom-tailored treatment increases the chance of therapeutic success. Health care for obese patients, regardless of gender, should be carried out by a multispecialty therapeutic team consisting of a physician, educator, dietitian, psychologist, and physiotherapist. To effectively treat obesity, after achieving the goals of weight reduction and mitigation of obesity-related complication, considering the risk of obesity recurrence, a long-term follow-up is strongly recommended to consolidate the results.

### 6.1. Diet and Lifestyle Interventions

The cornerstone of weight management are lifestyle interventions, including dietary changes and physical activity. The recommended individual caloric deficit should lead to a reduction of 5–10% of body weight within 3–6 months of the therapy initiation. Weight loss via caloric restriction is usually the first-line recommendation for obesity treatment. Even though numerous studies have documented the beneficial effects of caloric restriction in inducing weight loss and slowing down the progression of multiple metabolic disorders, medical nutrition therapy, or MNT for short, alone is associated with limited efficacy. Moreover, difficulties with weight loss maintenance arise with the passing of time. It has been established that the challenges in maintaining weight reduction are due to counter-regulatory neuroendocrine network actions that promotes weight regain by affecting hunger and satiety mechanisms and decreasing energy expenditure [92,93].

Aerobic physical activity (30–60 min of moderate to vigorous intensity most days of the week) should be recommended in order to achieve weight loss, reduction in visceral adipose tissue, and favor weight maintenance after achieving initial weight loss. Regular physical activity, irrespective of weight loss, can improve many cardiometabolic risk factors, health-related quality of life, mood disorders, and body image disorders in adults with overweight or obesity [94]. Pharmacotherapy in obesity management may provide a beneficial adjunct to nonpharmacological approaches and facilitate achieving and maintaining healthy behaviors that, in the long haul, are necessary in the therapeutic process.

### 6.2. Pharmacotherapy of Obesity

The US Food and Drug Administration (FDA) and European Medicines Agency (EMA) have approved pharmacotherapy with proven effects of clinically significant weight loss as an adjunct therapy to lifestyle interventions. According to guidelines, pharmacotherapy aimed at weight reduction is recommended for patients with BMI ≥ 30 kg/m^2^ or ≥27 kg/m^2^ with obesity-related comorbidities, which increases cardiovascular risk, regardless of the patients’ gender.

In the history of pharmacotherapy of obesity, many promising substances have been used. However, due to either their low effectiveness or unacceptable safety profile, most have been withdrawn from the market or are intended for short-term use only [95]. FDA guidelines accounted for, a drug is considered effective in the treatment of obesity if it results in at least 5% average weight loss and when more than 35% of patients achieve a weight loss of at least 5% after one year of using the drug. This translates into measurable lowering of cardiovascular risk [94]. Currently, five drugs are approved for weight-reduction therapy (orlistat, naltrexone/bupropion, 3 mg liraglutide, 2.4 mg semaglutide, phentermine/topiramate).

Orlistat, by inhibiting lipases in the gastric, small intestine, and pancreatic mucosa, limits the breakdown of triglycerides into free fatty acids and, consequently, decreases their absorption in the intestines. Its effect on weight reduction is more effective than a low-fat diet. Its use also contributes to lower insulin resistance, fasting plasma glucose, low-density lipoprotein cholesterol, and systolic and diastolic blood pressure [96].

Naltrexone ER/bupropion ER is a combination drug. Bupropion is a neuronal dopamine and norepinephrine reuptake inhibitor and a nicotinic receptor antagonist. It is used alone (that is without naltrexone) in the treatment of depression and nicotine dependency. Naltrexone is an opioid antagonist used in the treatment of opioid and alcohol addiction. In the treatment of obesity, bupropion inhibits food intake by exerting its action on the reward system. It activates the appetite-reducing neuropeptide proopiomelanocortin (POMC) and causes dopamine activation, which is pathologically lowered in obese patients. Naltrexone, on the other hand, inhibits the appetite-increasing effect of beta-endorphins caused by the activation of the cannabinoid-1 receptor. It also acts synergistically with bupropion; naltrexone, through antagonism of endogenous opioids, enhances the appetite suppression caused by POMC [97].

Incretin drugs are a class of drugs consisting of glucagon-like peptide-1 (GLP-1) analogues. These are substances that mimic the effects of native, endogenous GLP-1, a naturally occurring substance that promotes weight loss by reducing energy intake, decreasing hunger, and increasing satiety. GLP-1 analogues exhibit a peripheral and central mechanism of action. Their central mechanism of action is mediated through GLP-1 receptors in the hypothalamus, reducing the feeling of appetite. This mechanism is based on the stimulation of transcriptional neurons regulated by POMC and CART (cocaine and amphetamine related peptide), which suppress appetite and indirectly inhibit the protein neurons associated with the NPY (neuropeptide Y) and AgRP (agouti-related protein), neuropeptides responsible for the feeling of hunger [98]. Studies have shown that GLP-1 receptors in the pancreas and brain are responsible for the corresponding improvement in glycemic control and body weight. Among many GLP-1 agonists approved for the treatment of type 2 diabetes mellitus, only liraglutide 3.0 mg once-daily and semaglutide 2.4 mg once-weekly have been approved for obesity management. Studies have shown that both liraglutide and semaglutide also exert beneficial effects on cardiovascular risk, although the exact mechanism of this effect is not well established and is still under scrutiny [99,100].

A very promising new molecule in the pharmacotherapy of obesity is tirzepatide, a novel dual glucose-dependent insulinotropic polypeptide (GIP) and GLP-1 receptor agonist (RA), which demonstrated substantially greater glucose control and weight loss when compared with selective GLP-1RA dulaglutide and semaglutide [101,102,103]. Tirzepatide combines the actions of GLP-1 (reducing appetite, enhancing glucose-dependent insulin secretion) and glucagon (promoting gluconeogenesis) receptors while also activating GIP (increasing insulin secretion) and shown promising results in clinical trials, leading to significant weight loss and improvements in glycemic control in patients with type 2 diabetes and obesity. As with other GLP-1 receptor agonists, tirzepatide may cause gastrointestinal side effects, such as nausea, vomiting, and diarrhea.

A real breakthrough in the pharmacological treatment of obesity has turned out to be triple hormone receptor agonist, retatrutide, which activates GLP-1, GIP, and glucagon receptors and promotes spectacular weight reduction in clinical trials [104]. By activating three types of receptors, retatrutide reduces appetite, increases insulin secretion, and reduces glucose production. The drug’s efficacy was similar to that of bariatric-metabolic surgery and the majority of participants receiving the 12-mg dose had lost 20% or more of their baseline weight at 48 weeks and 26% had lost 30% or more of their original weight [105]. As with any medication, triple G receptor agonists may have side effects, including gastrointestinal symptoms such as nausea and vomiting. Long-term safety data are still limited, and further research is needed to fully understand their risk-benefit profile.

Phentermine/topiramate CR is a combination drug. Phentermine suppresses the appetite by increasing epinephrine secretion in the hypothalamus. Topiramate, a gamma-aminobutyric acid agonist, a glutamate antagonist, and a carbonic anhydrase inhibitor, is used alone in the treatment of epilepsy and prevention of migraines. In the treatment of obesity, it is used in lower doses and allows weight reduction through an effect synergistic with phentermine on the feeling of satiety [106].

The selection of the appropriate weight-reducing pharmacotherapy requires a careful analysis of the cognitive and behavioral causes of the patient’s eating disorders in order to optimally classify the patient groups and achieve the best treatment results.

There are still many promising therapies in clinical trials. Melanocortin receptor agonists in the hypothalamus, such as setmelanotide, are approved by FDA in patients six years and older with obesity due to three rare genetic conditions: proopiomelanocortin (POMC) deficiency, proprotein subtilisin/kexin type 1 (PCSK1) deficiency, and leptin receptor (LEPR) deficiency confirmed by genetic testing.

It is worth mentioning that cellulose and citric acid in an oral, non-systemic, superabsorbent hydrogel are used in conjunction with diet and exercise to aid weight management in obese or overweight adults. The drug contains 2 mm particles that swell in the stomach/small intestine after drinking water thus causing a feeling of satiety. First observations are very promising. New therapies have been shown to be effective, safe, and well-tolerated in clinical studies. However, further research is required to determine their long-term effectiveness and safety profile in the treatment of obesity. A summary of obesity pharmacotherapy is presented in Table 1.

In summary, pharmacotherapy for obesity is a valuable option for patients who are unable to lose weight and maintain achieved weight loss through behavioral changes alone. This treatment option should also be highly recommended for those who do not meet the eligibility criteria for bariatric surgery or who failed to maintain weight loss following bariatric procedures.

### 6.3. Bariatric Surgery

Bariatric surgery complements the therapy for all patients with severe obesity, i.e., BMI ≥ 40 kg/m^2^ and the patients with BMI ≥35 kg/m^2^ and obesity-associated comorbidities [107] after non-surgical methods of treatment have failed. There is a great body of evidence for the metabolic improvement of numerous diseases and disorders in obese patients after undergoing bariatric surgery. The probability of achieving normal weight or maintaining 5% body weight reduction with only non-surgical interventions is very low [108]. Fildes et al. reported that for severely obese men with BMI between 40.0–44.9 kg/m^2^, the annual likelihood of attaining normal body weight was 1 in 1290, compared to 1 in 677 for women with morbid obesity. Furthermore, the annual probability of achieving a 5% weight reduction was 1 in 8 for men and 1 in 7 for women with morbid obesity [108]. These observations indicate that bariatric procedures should not be delayed in patients with advanced and complicated obesity. However, it is difficult to draw firm conclusions due to fact that in studies, men represent only a minority among patients who undergo bariatric surgery [109]. In a multicenter analysis of more than 60,000 patients who undergone bariatric surgery in the US, it was observed that male patients lost less weight and had a smaller decrease in comorbidities and had more complications when compared to women. Moreover, when compared to females, male patients were older on average, had a higher BMI, and more comorbidities, which indicates that they probably sought medical attention much later in the course of the disease than women did [110]. Curiously, satisfaction with the surgical procedure was reported to be greater in men. An improvement in the quality of life after bariatric surgery was confirmed in both sexes, men and women.

Among the many available surgical methods, such as Roux-en-Y gastric bypass, biliopancreatic diversion/duodenal switch, sleeve gastrectomy, or laparoscopic adjustable gastric banding (LAGB), all seem to bring positive results depending on the experience of the operator [107,111]. When qualifying a patient for a specific procedure, it should be remembered that, in simplified terms, less invasive procedures such as LAGB, vagal blocking, and endoscopic procedures are usually associated with lower weight loss. However, they are also characterized by significantly lower perioperative and long-term complications and risks.

For instance, protein malnutrition is a quite common consequence of biliopancreatic diversion (3–18%) and distal Roux-en Y gastric bypass (RYGB) with a Roux limb ≥150 cm (13%). On the other hand, the frequency of this complication is significantly lower after RYGB with a Roux limb <150 cm (<5%). Dumping syndrome is most characteristic after gastric bypass surgery (70–75% of patients) and also after sleeve gastrectomy (40% of patients) [112].

Malabsorption results in vitamin D deficiency, a common occurrence despite routine prophylactic supplementation with cholecalciferol. Vitamin D deficiency has high incidence after biliopancreatic diversion, gastric bypass, and omega-loop gastric bypass, generally in the range of 50–60% of patients, depending on the method used. The safest procedures in this regard are a sleeve gastrectomy or adjustable gastric band, which are associated with only minimal risk of vitamin D deficiency [112].

Regardless of the surgical procedure used, studies have shown that in the postoperative period, mainly due to the loss of visceral fat and changes in intestinal peptides, there is a significant improvement in metabolic parameters: lipid profile, glycemic control, reduction of insulin resistance, MAFLD (metabolic associated fatty liver disease), inflammation, sleep apnea, or stricter blood pressure control [113,114,115,116,117,118,119]. These benefits were assessed in both shorter and longer follow-up periods, which allowed us to observe that some of them are transient. In these cases patients require further monitoring and care, including permanent lifestyle changes and indefinitely using weight-reducing medications [116]. Besides direct impact through weight reduction, bariatric surgery allows for us to improve the quality of life and to reduce the incidence of civilization diseases and all-cause mortality due to the improvements of many health indicators in the postoperative period [120,121,122].

## 7. Conclusions

Although obesity is a chronic disease with no spontaneous remission and with a tendency for relapse, it is preventable, treatable and a potentially reversible cause of numerous metabolic disorders. The need for an all-encompassing adult-oriented approach for diagnosis and treatment has not been fully addressed. Obesity management includes early diagnosis, establishment of obesity-related complications, and selection of optimal, individualized therapeutic strategies. Careful attention should be paid to the population of males with obesity who tend to seek medical attention with a considerable delay when compared to females with obesity. Furthermore, male obesity is usually more exacerbated and complicated with more comorbidities and higher resultant mortality at the time of diagnosis. An additional, often not appreciated enough, challenge in obesity management is the necessity to engage the patient in the therapeutic process. This requires constant and repeated education as well as a supportive, multispecialty team to enhance the patient’s motivation.

Many countries worldwide are facing numerous challenges in obesity prevention and treatment to ever growing extent. Healthcare systems should take a more proactive role in this important area. It is recommended to oblige health care providers to screen for obesity and its complications, as well as to actively participate in its management from the earliest stages of the disease. People with obesity should have access to evidence-based interventions, including medical nutrition therapy, physical activity, psychological interventions, pharmacotherapy, and metabolic surgery.

## Figures and Tables

**Table 1:** Drugs used in the treatment of obesity [94,95,96,97,98,99,100,101,106,107,108].

Drug	Mechanism of Action	Dosage	Average Weight Loss	Side Effects	Additional Benefits
Orlistat	Pancreatic and gastric lipase inhibitor	120 mg three times daily with meals	Around 5–10% of initial body weight	Gastrointestinal side effects (diarrhea, bloating flatulence)	Improves lipid profile; low risk of systemic side effects
Phentermine /Topiramate	Combination of sympathomimetic amine, anorectic and antiepileptic drug	Varies based on titration schedule	Around 5–10% of initial body weight	Dyspepsia, insomnia, constipation, dry mouth, transient peripheral neuropathy	Improves glycemic control; reduces blood pressure
Bupropion /Naltrexone	Combination of antidepressant and an opiod receptor antagonist, affecting appetite and reward pathways in hypothalamus	Gradual titration to target dose (varies, maximal daily dose bupropion 360 mg/32 mg naltroxone)	Around 5–10% of initial body weight	Nausea, headache, constipation, insomnia, dry mouth	May improve mood and emotional eating behaviors
Liraglutide	GLP-1 receptor agonist	Once daily sc. injection, 3.0 mg	Around 5–10% of initial body weight	Nausea, diarrhea, vomiting, headache, constipation	Improves glycemic control; reduces cardiovascular risk
Semaglutide	GLP-1 receptor agonist	2.4 mg once weekly s.c.	Around 15–20% of initial body weight	Nausea, diarrhea, vomiting, headache, constipation	Improves glycemic control; reduces cardiovascular risk
Tirzepatide	Dual GIP/GLP-1/receptor agonist	Once-weekly s.c injection, 15 mg	Around 13–20% of initial body weight	Nausea, diarrhea, vomiting, headache, constipation	Improves glycemic control; cardiovascular risk under investigation
